# Changes of Quality of Life after Gastric Tube Reconstruction in Adenocarcinoma of the Esophagogastric Junction

**DOI:** 10.12669/pjms.295.3879

**Published:** 2013

**Authors:** Chaoyong Shen, Hongxin Yang, Bo Zhang, Haining Chen, Zhixin Chen, Jiaping Chen

**Affiliations:** 1 Chaoyong Shen, Department of Gastrointestinal surgery, West China Hospital, Sichuan University, Chengdu 610041, Sichuan Province, China.; 2 Hongxin Yang, Department of Gastrointestinal surgery, West China Hospital, Sichuan University, Chengdu 610041, Sichuan Province, China.; 3 Bo Zhang, Department of Gastrointestinal surgery, West China Hospital, Sichuan University, Chengdu 610041, Sichuan Province, China.; 4 Haining Chen, Department of Gastrointestinal surgery, West China Hospital, Sichuan University, Chengdu 610041, Sichuan Province, China.; 5 Zhixin Chen, Department of Gastrointestinal surgery, West China Hospital, Sichuan University, Chengdu 610041, Sichuan Province, China.; 6 Jiaping, Chen. Department of Gastrointestinal surgery, West China Hospital, Sichuan University, Chengdu 610041, Sichuan Province, China.

**Keywords:** Adenocarcinoma of esophagogastric junction, Gastric tube, Quality of life, EORTC QLQ-C30, EORTC QLQ-STO22

## Abstract

***Objective:*** To investigate changes of quality of life (QOL) of patients with adenocarcinoma of the esophagogastric junction (AEG) after gastric tube anastomosis.

***Methods:*** From January 2009 to December 2011, eighty-seven patients with Types II and III AEG were selected for gastric tube reconstruction after proximal gastrectomy. The QOL of the patients was assessed using the Chinese versions of the EORTC QLQ-C30 and the EORTC QLQ-STO22 preoperatively, as well as one and two years postoperatively.

***Results:*** The QLQ-C30 showed that the global health of the respondents decreased at one year after the surgery (P=0.02). The preoperative score for physical function was significantly better than the one- and two-year post-operation scores. The preoperative scores for pain, nausea and vomiting, and economic difficulties were worse than the one- and two-year post-operation scores (P<0.05). Diarrhea was worse at one year post-operation than during pre-operation (P = 0.00), but improved at two years after the operation. The QLQ-STO22 scales showed that the preoperative dysphagia score was better than one-year post-operation, and no significant differences were observed in terms of dysphagia between the pre-operation and two-year postoperative periods. Preoperative reflux and taste scores were better than those after the operation (P<0.05). The hair loss score at one-year post-operation was worse than at either pre-operation or two-year post-operation.

***Conclusions: ***Most QOL scales worsened after surgery, particularly at postoperative year one. However, the scales can be gradually recovered to preoperative levels. The physical function, nausea and vomiting, reflux, taste, and financial difficulties did not fully recover two years after the operation.

## INTRODUCTION

The adenocarcinoma incidence of the esophagogastric junction (AEG) has evidently increased in recent years.^1^^,^^2^ The prognosis of patients with AEG, however, is still poor, largely as a result of most patients being diagnosed at an advanced stage. AEG is currently divided into three types according to Siewert’s classification.^3^ Total or proximal gastrectomy has been recommended for Type II or Type III AEG, with direct anastomosis between the remnant stomach and the esophagus being recommended after proximal gastrectomy. However, direct anastomosis is followed by some severe complications, such as gastrointestinal reflux and reflux esophagitis. To reduce such postoperative complications, researchers have proposed a new technique, namely, gastric tube reconstruction, which involves shaping the gastric remnant into a tube after proximal gastrectomy followed by anastomosis.^4^^,^^5^ Researchers have concluded that this technique can reduce the number of complications at the early postoperative stage. However, to our knowledge, very little research has addressed the longitudinal changes of QOL resulting from this new digestive tract reconstruction procedure.

With the increasing concern regarding postoperative recovery, we need to examine the available information needs and sources regarding cancer patients after surgery.^6^ QOL measurement provides relevant information on long-term postoperative cancer survival. With the increasing overall survival rate for gastric cancer patients, physicians have become more interested in the QOL of gastric cancer patients. A number of questionnaires, such as the Spitzer index, the SF-36 health survey questionnaire, the European Organization for Research and Treatment of Cancer Quality of life Questionnaire Core-30 (EORTC QLQ-C30), and the EORTC QLQ-STO22 (stomach module) are widely used for QOL measurement.^7^^-^^10^ Of these questionnaires, the QLQ-C30 is usually used in combination with the stomach module to evaluate gastric cancer patients’ QOL because these questionnaires can obtain more concrete and valuable information. These two QOL questionnaires have already been converted into different versions.^11^^,^^12^

In this study, we used the Chinese version of the EORTC QLQ-C30 and QLQ-STO22 to evaluate the QOL of Type II and Type III AEG patients who had undergone the new gastric tube reconstruction after proximal gastrectomy.

## METHODS

***Participant Recruitment:*** The protocol was approved by the institutional review board and committee of West China Hospital of Sichuan University. All the patients gave their written consent to undergo the procedure. We analyzed the data obtained from patients with Type II or Type III AEG who underwent curative proximal gastrectomy with gastric tube reconstruction between January 2009 and December 2011 at the Department of Gastrointestinal Surgery, West China Hospital, Sichuan University. All patients underwent laparotomy. Age, gender, tumor diameter (cm), hospital stay (days), histological type, Siewert type, Borrmman type, depth of invasion (T), lymph node metastasis (N), and tumor TNM stage^13^ were obtained from the patients’ medical records (Table-I).

**Table-I:** Characteristics of patients enrolled in this study

*Clinical feature*	*Numbers of patients (n=87)*	*Percentage (%)*
Gender		
Male	66	75.9
Female	21	24.1
Age (years)	62.48±8.25	
Total hospital stay (days)	19.17±4.76	
Tumor diameter (cm)	4.05±1.67	
Number of removed lymph nodes	21.55±8.16	
Number of positive nodes	3.91±6.16	
Depth of invasion		
T1	4	4.6
T2	15	17.3
T3	21	24.1
T4	47	54.0
Lymph node metastasis		
N0	24	27.6
N1	27	31.0
N2	21	24.1
N3	15	17.3
Distant metastasis		
M0	87	100
M1	0	0
Tumor TNM stage		
I	10	11.5
II	25	28.7
III	52	59.8
IV	0	0
Histological type		
Well differentiated	1	1.1
Well-moderately differentiated	2	2.3
Moderately differentiated	27	31.0
Moderate-poorly differentiated	31	35.7
Poorly differentiated	26	29.9
Siewert type		
II	40	46.0
III	47	54.0
Borrmman type		
I	12	13.8
II	55	63.2
III	20	23.0
IV	0	0

***Inclusion criteria:*** (1) Patients diagnosed with AEG (Siewert Type II/III) through gastroscope biopsy and upper gastrointestinal barium examination. (2) No clear proof of distant metastasis or adjacent tissue invasion shown by routine preoperative examination, such as chest radiography, abdominal CT scan, or endoscopic ultrasonography. Exclusion criteria: Patients with evidence of recurrence, metastasis, or death during the follow-up period were excluded from this study. Accordingly, 87 patients with Type II or Type III AEG who had undergone curative surgery were enrolled in this study. All of the patients or their family members were informed of the new technique and the follow-up for postoperative QOL measurement. 

**Fig.1:**
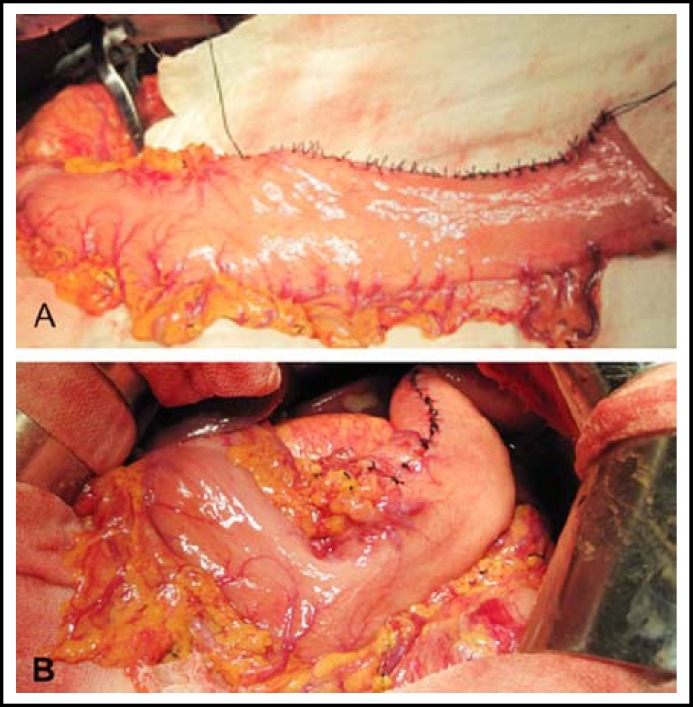
Technique for gastric tube reconstruction. The remant stomach after proximal gastrectomy was finally made into an approximately 4-5 centimeters in width and 30 centimeters in length tube by linear cut staplers (A). An antomosis was made between the rear wall of gastric tube and the esophagus (B).

***Operation Procedure:*** The surgical procedure was started with a midline upper abdominal incision under general anesthesia. Proximal gastrectomy and distal esophagectomy were then performed through this incision. All patients underwent standard treatment according to the criteria of the Japanese Gastric Cancer Association (JGCA), and the proximal gastrectomy, vagectomy, and D2 or greater D2 lymphadenectomy were included. The remnant stomach volume was approximately 40% to 70% of its original size. Linear-stapling devices were used to shape the lesser curvature that lay at approximately 3 cm above the pylorus of the gastric remnant into a tube with approximately 4 cm to 5 cm in width and 30 cm in length (Fig.1). The rear wall of the gastric tube was then anastomosed to the esophagus using a stapler, thereby completing the digestive tract reconstruction. All operations were performed by the same skilled medical team.

***Data Collection and Assessment of QOL: ***Patients or their family members were asked to complete the questionnaires (Chinese version of EORTC QLQ-C30 and QLQ-STO22) preoperatively, at one post-operation, and at two years’ post-operation.

The EORTC QLQ-C30 is composed of five functional scales (physical, role, cognitive, emotional, and social), three symptom scales (pain, fatigue, and nausea and vomiting), six single items (dyspnea, appetite loss, sleep disturbance, constipation, diarrhea, and financial difficulties), and a global health status^9^ The EORTC QLQ-STO22 is composed of five multi-item scales (dysphagia, pain, reflux, eating, and anxiety) and four single items (dry mouth, tasting, body image, and hair loss).^10^ Once all of the scales were scored, the raw scores for each scale were linearly transformed to scores ranging from 0 to 100, according to the Scoring Manual. For the global health status and functional scales in the QLQ-C30, a high score represents a better quality of life. However, high scores in the symptom scale and single items represent poor quality of life. For the QLQ-STO22, a high score represents low quality of life.

*Statistical Analysis: *Quantitative data were expressed as means ± SD, repeated measured ANOVA was used to compare the score of each scale before and after surgery. *P*<0.05 was considered statistically significant. Statistical analysis was conducted using SPSS 17.0 for Windows.

## RESULTS

***Clinical Outcomes:*** Of the 87 patients, 16 were lost during the follow-up, 17 cases died, and 8 patients experienced tumor recurrence or distant organ metastasis during the follow-up period. Finally, we got 71 and forty-two eligible QOL questionnaires at one post-operation, and at two years’ post-operation, respectively. All patients completed the QOL questionnaires preoperatively.

***EORTC QLQ-C30:*** The global health status of EORTC QLQ-C30 decreased at one year after surgery when compared with pre-operation (P = 0.02). However, no statistically significant difference was observed between pre-operation and two-year postoperative status (P = 0.76). The preoperative physical function scores were significantly better than those at one and two years’ post-operation, indicating that the patients had poor physical fitness after their operations. The preoperative scores for pain, nausea and vomiting, and economic difficulties were worse than either the one- or two-year post-operation scores (P < 0.05). No statistical differences were observed between the one- and two-year post-operation scores for these three scales. The diarrhea score increased in the one-year post-operation group (P = 0.00), but decreased two years after surgery. However, no statistically significant differences were observed before and after the operation in terms of role function, emotional function, social function, cognitive function, fatigue, and so on, during the study period. The score for all scales are shown in Table-II.

**Table-II:** QOL scores as measured by the Chinese version of the EORTC QLQ-C30

				*P*		
*Variables*	*Pre-operation (a)*	*1-year post-operation (b)*	*2-year post-operation (c)*	*a:b*	*a:c*	*b:c*
Global QOL	59.0±10.8	54.3±13.5	59.7±13.6	0.02	0.76	0.04
Physical function	88.0±12.2	77.3±16.2	78.8±16.8	0.00	0.00	0.63
Role function	72.0±16.2	69.5±16.2	71.4±14.3	0.33	0.85	0.57
Emotional function	74.2±12.0	72.6±16.7	73.0±16.5	0.50	0.70	0.88
Physical function	88.0±12.2	77.3±16.2	78.8±16.8	0.00	0.00	0.63
Role function	72.0±16.2	69.5±16.2	71.4±14.3	0.33	0.85	0.57
Social function	78.1±13.8	74.0±15.1	76.6±14.1	0.08	0.60	0.39
Congnitive function	89.2±12.4	88.9±11.8	89.0±12.1	0.87	0.92	0.97
Fatigue	18.1±15.2	22.1±16.9	21.9±17.0	0.13	0.25	0.96
Pain	28.9±22.0	15.5±17.3	13.8±15.9	0.00	0.00	0.66
Nausea and vomiting	6.3±14.1	17.8±14.9	13.8±15.4	0.00	0.01	0.19
Appetite loss	28.7±20.4	30.6±21.1	25.7±21.5	0.57	0.47	0.25
Constipation	7.7±18.1	7.6±15.1	8.6±16.8	0.99	0.79	0.79
Diarrhea	5.4±14.2	14.0±19.9	12.4±19.9	0.00	0.05	0.67
Sleep disturbance	26.4±23.3	31.1±20.1	28.5±24.4	0.20	0.64	0.59
Dyspnea	14.9±18.1	14.4±18.3	18.1±24.7	0.87	0.42	0.36
Financial difficulties	23.7±22.1	40.1±25.2	33.3±19.8	0.00	0.04	0.15

***EROTC QLQ-STO22:*** In the EORTC QLQ-STO22, the patients scored worse for dysphagia at one year after surgery (P = 0.00) than during pre-operation, whereas no statistically significant difference was observed between the pre-operation and two-year post-operation scores (P = 0.28). The eating restriction score significantly increased at one year post-operation (P < 0.05), but decreased at two years after surgery, with no statistically significant difference from the preoperative score (P = 0.17). Changes observed, such as those in the QLQ- C30 pain scale, indicated that surgery or other postoperative treatment can alleviate stomach pain (P < 0.05). The preoperative reflux and taste scores were better than those of post-operation (P < 0.05). The hair loss score was worse at one year after surgery, possibly as a result of the post-surgery chemotherapy. No statistically significant difference was observed in terms of anxiety or dry mouth scale before and after the surgery (Table-III).

**Table-III:** QOL scores as measured by the Chinese version of the EORTC QLQ-STO22

				*P*		
*Variables*	*Pre-operation(a)*	*1-year post-operation(b)*	*2-year post-operation(c)*	*a:b*	*a:c*	*b:c*
Dysphagia	20.2±18.1	29.1±17.2	24.1±20.2	0.00	0.28	0.18
Pain	29.8±12.1	25.7±10.9	23.5±14.2	0.03	0.01	0.40
Reflux	12.1±15.3	23.4±14.3	19.3±12.4	0.00	0.01	0.17
Eating	17.1±12.9	24.6±11.2	20.5±10.7	0.00	0.17	0.09
Anxiety	30.2±13.9	33.5±15.6	32.7±15.9	0.18	0.42	0.80
Dry mouth	10.0±15.3	13.1±21.9	8.6±14.8	0.28	0.70	0.23
Taste	10.7±16.5	36.0±26.3	32.3±23.5	0.00	0.00	0.42
Body image	37.5±20.2	39.6±18.0	31.4±18.0	0.49	0.11	0.04
Hair loss	11.9±18.3	8.5±24.7	7.6±14.2	0.04	0.30	0.01

## DISCUSSION

Studies on the long-term QOL assessment of gastric tube reconstruction after proximal gastrectomy are limited. The current study has shown that most scales worsened at one year after the procedure, but some scales recovered to preoperative levels at two years after the procedure. However, physical function, nausea and vomiting, reflux, taste, and financial difficulties did not fully recover during the two-year period.

AEG patients who undergo proximal gastrectomy have their anatomic anti-reflux barriers destroyed. Meanwhile, gastric acid secretion does not completely abate,^14^ which may result in severe postoperative regurgitation or heartburn, which in turn affects the normal life of the patients. Shiraishi et al.^15^ first proposed a technique for proximal gastrectomy involving gastric tube reconstruction, which reduced heartburn and regurgitation. Chen et al^4^ and Adachi et al^5^ showed that this surgical procedure can effectively reduce postoperative gastroesophageal reflux and reflux esophagitis during the one-year follow-up. However, insufficient research has been done to assess the long-term QOL effects of this new technique. The EORTC designed the QLQ-C30 (for patients with cancer) and QLQ-STO22 (a gastric cancer-specific module) in 1993 and 1998, respectively. In this study, we used the Chinese version of the EORTC QLQ-C30 and QLQ-STO22,^16^^,^^17^ to assess QOL.

Using EORTC QLQ-C30, past studies have observed that most functional scales recover to preoperative levels at 6 to 12 months after operation.^18^^,^^19^ In the present study, many functional scales and global health status scores at one year post-operation are worse than the pre-operation and two-year post-operation scores. Some potential correlative factors of QOL such as education level, marriage, employment status, and poor lifestyle habits (smoking, drinking), may make a difference in QOL. Moreover, we notice that most AEG patients (Type II/III) were diagnosed at advanced or terminal stage, and these patients may take a longer time to recovery. We have found no obvious differences in the preoperative and one-year postoperative social and cognitive function scores, indicating that the patients recovered to baseline status at one year post-operation. 

These results are consistent with those of previous studies.^18^ Many patients suffered from postoperative diarrhea because of denervation resulting from truncal vagotomy. Kobayashi et al.^20^ showed that postoperative diarrhea does not fully recover during the first year of progress. In the present study, the diarrhea score was worse at one year after surgery, but recovered one year later, indicating that diarrhea resulting from proximal gastrectomy improved to preoperative levels at two years after surgery. The scales for financial difficulties and nausea/vomiting did not fully recover at two years after the operation. Zhou et al.^21^ pointed out that these two scales cannot recover to the level of healthy people at three years’ post-operation.

Moreover, a lengthier investigation in Korea observed that gastric cancer patients who had undergone a curative distal subtotal gastrectomy still suffered from financial difficulties and nausea/vomiting on their fifth annual follow-up visit. Perhaps a return to baseline will take a much longer time. Thus, it is essential to conduct further follow-ups to verify whether or not the patients can fully recover or not. Kong et al.^18^ found no statistically significant difference in the pain scale at pre- and post-operation, attributing this peculiar result to pain score reaching the maximum level rapidly and recovering quickly before the first postoperative questionnaire survey is conducted. By contrast, the patients enrolled in the present study exhibited quite a different pattern. All of the respondents reported substantial pain relief at one and two years after surgery. 

Hence, it is reasonable and logical to assume that patients with medium or advanced cancers often experience more pain than early cancer patients. In addition, this study involved more patients at advanced TNM stages in that of Kong et al. Thus, the baseline pain score in this study was much higher. However, the pain score in our study was not much different from that of Kong et al. at one year after surgery, which may be the reason for the significant pain relief after operation exhibited in the present study.

For EORTC QLQ-STO22, the dysphagia, eating restriction, and hair loss scales recovered to baseline within two years. However, reflux and taste did not fully recover at two years’ post-operation. Another investigation showed a marked difference between gastric cancer patients and the healthy population in terms of reflux scale at five years’ post-operation.^22^ This result may be the adverse outcome of gastrectomy; however, different surgical procedures may have different effects on recovery time and mode. Patients’ decreased stomach pain after surgery should be attributed to the removal of the tumor and rational postoperative treatment, such as postoperative chemotherapy or supportive treatment. No difference was observed between the pre-operation and post-operation anxiety scores. The patients may have had a degree of concern about their diseases or the potential risks of operation prior to surgery. They are also worried about their future health on account of specific postoperative complications and discomforts, which could explain why there are no significant differences in the anxiety scale during the study. Moreover, the scores of dry mouth and body image scales at one year after surgery were slightly poorer than those prior to the operation, but did not reach statistical significance. This result indicates that patients had reverted to the baseline for these two scales one year post-operation.

Some scales varied in recovery time, which may be associated with different surgical procedures, methods of anastomosis, tumor location, and application of postoperative chemotherapy. The type of surgery can also provide more information to a surgeon or patient in the future. Therefore, we plan to explore the relationship between QOL and these factors in future studies.

## CONCLUSION

This study showed that patients with Type II or Type III AEG who underwent gastric tube reconstruction after proximal gastrectomy experience problems involving nausea and vomiting, reflux, taste, and financial difficulties. These differences exist for at least two years. However, most scales worsened after surgery, and then recovered to preoperative levels. For the scales that did not recover over the course of the study, further follow-ups would be carried out to confirm whether or not the patients can return to baseline or not. Accordingly, countermeasures should be taken to treat these discomforts and improve the QOL of the patients.
